# AT-101, a small molecule inhibitor of anti-apoptotic Bcl-2 family members, activates the SAPK/JNK pathway and enhances radiation-induced apoptosis

**DOI:** 10.1186/1748-717X-4-47

**Published:** 2009-10-23

**Authors:** Shuraila F Zerp, Rianne Stoter, Gitta Kuipers, Dajun Yang, Marc E Lippman, Wim J van Blitterswijk, Harry Bartelink, Rogier Rooswinkel, Vincent Lafleur, Marcel Verheij

**Affiliations:** 1 Department of Radiation Oncology, The Netherlands Cancer Institute - Antoni van Leeuwenhoek Hospital, Amsterdam, The Netherlands; 2 Department of Radiation Oncology, VU University Medical Center, Amsterdam, The Netherlands; 3 Ascenta Therapeutics, Inc., Malvern, Pennsylvania, USA; 4 Department of Internal Medicine, University of Michigan Health System, Ann Arbor, Michigan, USA; 5 Division of Immunology, The Netherlands Cancer Institute - Antoni van Leeuwenhoek Hospital, Amsterdam, The Netherlands

## Abstract

**Background:**

Gossypol, a naturally occurring polyphenolic compound has been identified as a small molecule inhibitor of anti-apoptotic Bcl-2 family proteins. It induces apoptosis in a wide range of tumor cell lines and enhances chemotherapy- and radiation-induced cytotoxicity both *in vitro *and *in vivo*. Bcl-2 and related proteins are important inhibitors of apoptosis and frequently overexpressed in human tumors. Increased levels of these proteins confer radio- and chemoresistance and may be associated with poor prognosis. Consequently, inhibition of the anti-apoptotic functions of Bcl-2 family members represents a promising strategy to overcome resistance to anticancer therapies.

**Methods:**

We tested the effect of (-)-gossypol, also denominated as AT-101, radiation and the combination of both on apoptosis induction in human leukemic cells, Jurkat T and U937. Because activation of the SAPK/JNK pathway is important for apoptosis induction by many different stress stimuli, and Bcl-X_L _is known to inhibit activation of SAPK/JNK, we also investigated the role of this signaling cascade in AT-101-induced apoptosis using a pharmacologic and genetic approach.

**Results:**

AT-101 induced apoptosis in a time- and dose-dependent fashion, with ED_50 _values of 1.9 and 2.4 μM in Jurkat T and U937 cells, respectively. Isobolographic analysis revealed a synergistic interaction between AT-101 and radiation, which also appeared to be sequence-dependent. Like radiation, AT-101 activated SAPK/JNK which was blocked by the kinase inhibitor SP600125. In cells overexpressing a dominant-negative mutant of c-Jun, AT-101-induced apoptosis was significantly reduced.

**Conclusion:**

Our data show that AT-101 strongly enhances radiation-induced apoptosis in human leukemic cells and indicate a requirement for the SAPK/JNK pathway in AT-101-induced apoptosis. This type of apoptosis modulation may overcome treatment resistance and lead to the development of new effective combination therapies.

## Background

Modulation of apoptosis sensitivity has emerged as a promising strategy to increase tumor cell kill [1]. Apoptosis or programmed cell death is a characteristic mode of cell destruction and represents an important regulatory mechanism for removing abundant and unwanted cells during embryonic development, growth, differentiation and normal cell turnover. Radiation and most chemotherapeutic drugs induce apoptosis in a time- and dose-dependent fashion. Failure to eliminate cells that have been exposed to mutagenic agents by apoptosis has been associated with the development of cancer and resistance to anticancer therapy. Indeed, several oncogenes mediate their effects by interfering with apoptotic signaling or by modulation of the apoptotic threshold. Bcl-2 and Bcl-X_L _are important inhibitors of apoptosis and frequently overexpressed in a variety of human tumors [2-7]. Increased levels of Bcl-2 and Bcl-X_L _have been associated with radio- and chemoresistance and poor clinical outcome in various types of cancer [8-12]. In fact, among all genes studied to date in the NCI's panel of 60 human tumor cell lines, Bcl-X_L _shows one of the strongest correlations with resistance to cytotoxic anticancer agents [13]. Therefore, inhibition of anti-apoptotic Bcl-2 family members represents an appealing strategy to overcome resistance to conventional anticancer therapies. In recent years, several agents targeting the Bcl-2 family proteins have been developed [14]

**Figure 1:**
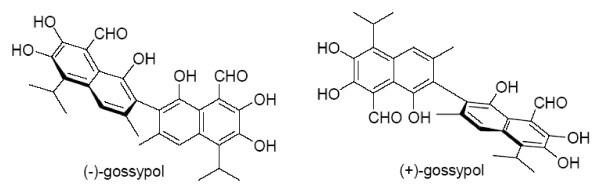
**Chemical structure of the (-) and (+) enantiomer of gossypol**.

Gossypol has been identified as a potent inhibitor of Bcl-X_L _and, to a lesser extent, of Bcl-2 [15]. It is a naturally occurring polyphenolic compound derived from cottonseed and was initially evaluated as an anti-fertility agent. Gossypol induces apoptosis in tumor cells with high Bcl-X_L _and/or Bcl-2 expression levels, leaving normal cells with low expression levels (*e.g*. fibroblasts, keratinocytes) relatively unaffected [16]. Racemic (±)-gossypol is composed of 2 enantiomers: (+)-gossypol and (-)-gossypol (Fig. 1). (-)-gossypol, also denoted as AT-101, binds with high affinity to Bcl-X_L_, Bcl-2 and Mcl-1 [17] and is a more potent inducer of apoptosis than (+)-gossypol [15,16,18]. AT-101-induced cell death is associated with apoptosis hallmarks like Bak activation, cytochrome c release and effector caspase 3 cleavage [19].

Few studies have addressed the effect of gossypol in combination with chemo- or radiotherapy [20-25]. *In vitro*, enhanced apoptosis and reduced clonogenicity was observed when AT-101 was combined with radiation in a prostate cancer line [22], while CHOP chemotherapy significantly enhanced AT-101-induced cytotoxicity in lymphoma cells [21]. Recent studies in multiple myeloma cell lines demonstrated synergistic toxicity with dexamethasone [25]. In head and neck squamous carcinoma cell lines the combination of stat3 decoy and AT-101 as well as the triple combination of erlotinib, stat3 decoy and AT-101 showed significant enhancement of growth inhibition [26]. Also *in vivo *the combined treatment of AT-101 with radiation [22] or chemotherapy [21] resulted in superior anti-tumor efficacy compared to single agent treatment. The interaction between radiation and AT-101 appeared to be sequence-dependent with radiation "sensitizing" the cells for AT-101, but not *vice versa *[22].

Activation of SAPK/JNK has been shown to play an important role in apoptosis induction by many stimuli, including radiation and chemotherapeutic drugs [27,28]. This, together with the observation that one of the major targets of AT-101, Bcl-X_L_, inhibits SAPK/JNK action [29] stimulated us to investigate whether gossypol activates this pathway and whether this contributes to the pro-apoptotic effect of this novel compound.

In the present study, we describe the apoptotic effect of ionizing radiation and AT-101 in the human leukemic cell lines U937 and Jurkat T. We determined whether the combination of both treatment modalities would induce higher levels of apoptosis than after single agent treatment and characterized the type of interaction. We also tested the hypothesis that activation of the SAPK/JNK pathway is important for AT-101-induced apoptosis in these cell systems.

## Methods

### Reagents

AT-101 was provided by Ascenta Therapeutics, Inc. (Malvern, PA, USA). (±)-Gossypol was purchased from Sigma-Aldrich. Stock solutions were prepared in dimethylsulfoxide to a concentration of 20 mM and stored at 4°C. Prior to use an aliquot was diluted in Dulbecco's modified Eagle's medium (DMEM; Invitrogen, Carlsbad, CA, USA). Phospho-SAPK/JNK (Thr183/Tyr185) monoclonal antibody was from Cell Signaling Technology, Inc. The SAPK/JNK inhibitor anthrax(1,9-cd)pyrazol-692H)-one (SP600125) [30] was obtained from BIOMOL Research Laboratories (Plymouth Meeting, PA, USA) and dissolved in dimethylsulfoxide.

### Cell culture and irradiation procedure

Human monoblastic leukemia cells (U937) and the human T lymphoid leukemic Jurkat cell line (J16, kindly provided by Prof. J. Borst, The Netherlands Cancer Institute, Amsterdam), both expressing Bcl-X_L_, Bcl-2 and Mcl-1 (not shown) were grown at a density between 0.1 × 10^6 ^and 1 × 10^6 ^cells/ml respectively in RPMI and Iscove's modified Dulbecco's medium (Invitrogen, Carlsbad, CA, USA, Paisley, Scotland), 8% heat-inactivated fetal calf serum, glutamine (2 mM), penicillin (50 U/ml) and streptomycin (50 μg/ml). U937 cells stably transfected with *TAM-67 *(U937/TAM-67 cells; a kind gift from dr. M.J. Birrer, National Cancer Institute, Rockville, Maryland) [31]. In selected experiments 2 human head and neck squamous cell carcinoma lines were used (VU-SCC-OE and UM-SCC-11B). These cell lines were grown in DMEM supplemented with 8% heat-inactivated fetal calf serum, glutamine (2 mM), penicillin (50 U/ml) and streptomycin (50 μg/ml). For irradiation experiments, cells were exposed to gamma rays from a ^137^Cs radiation source (Von Gahlen B.V., Didam, The Netherlands) at an absorbed dose rate of approximately 1 Gy/min. Control cells were sham-irradiated.

### Apoptosis assays

Apoptosis was determined by either staining with the DNA-binding fluorochrome *bis*benzimide (Hoechst 33258, Sigma) to detect morphological nuclear changes or by propidium iodide staining and FACScan analysis to determine the percentage of subdiploid apoptotic nuclei. For the *bis*benzimide staining, cells were washed once with PBS and resuspended in 50 μl of 3.7% paraformaldehyde. After 10 min at room temperature, the fixative was removed and the cells were resuspended in 15 μl of PBS containing 16 μg/ml *bis*benzimide. Following 15 min incubation, a 10 μl aliquot was placed on a glass slide, and 500 cells per slide were scored in duplicate for the incidence of apoptotic nuclear changes under a Olympus AH2-RFL fluorescence microscope using a UV1 exciter filter. For the propidium iodide staining, cells were seeded at 2 × 10^6 ^cells/ml, 200 μl/well in round-bottomed, 96-well microtiter plates. Cells were lysed in 200 μl Nicoletti Buffer (0.1% sodium citrate, 0.1% Triton X-100, and 50 μg/ml propidium iodide) and the percentage apoptotic nuclei, recognized by their subdiploid DNA content, was determined on a FACScan (Becton Dickinson, San Jose, CA) using Lysys II software.

### MTT assay

Cells were grown and treated in 96 well flat-bottomed plates. Cell survival was measured by spectrophotometrical quantification of the formation of blue formazan crystals which are formed when mitochondrial dehydrogenases in viable cells reduce 3-(4,5-dimethylthiazol-2-yl)-2,5-diphenyltetrazolium bromide (MTT; Sigma). To this end, treated cells were supplemented with 20 μl of MTT solution (5 mg/ml). After 15-30 min of incubation at 37°C the plates were centrifuged and the supernatant discarded. Formazan crystals were dissolved in 100 μl DMSO. Absorbance at 595 nm was measured using a Victor 2 absorbance reader (Perkin Elmer GMI, Inc, MN, USA).

### Western blotting

Western blot analysis was performed to detect activated SAPK/JNK. Cells were washed, replenished with serum free medium and left overnight. Subsequently, the cultures were treated with increasing doses of radiation and/or AT-101, washed and lysed in Triton lysis buffer (20 mM HEPES (pH 7.4), 2 mM EGTA, 50 mM, β-glycerophosphate, 1% Triton X-100, 2.5 mM MgCl_2_, 1 mM NA_3_VO_4_, 5 μM leupeptin, 2.5 μM aprotinin and 400 μM phenylmethylsulfonyl fluoride) on ice for 15 min. Lysates were clarified by centrifuging for 10 min at 3000 rpm, normalized for protein content and 80 μg of total lysate was loaded on Invitrogen 4-12% acrylamide NuPAGE novex bis-tris gels. Separated proteins were transferred to nitrocellulose membranes and blocked for 1 h with 5% (w/v) Nutrilon Premium (Nutricia Zoetermeer, The Netherlands) in TBS-T. Blots were probed with SAPK/JNK monoclonal antibody (1:500) in 5% Nutrilon in TBS-T. Control blots were probed with total SAPK/JNK polyclonal antibody (1:1000) in 1% Nutrilon in TBS-T. After secondary horseradish peroxidase-conjugated antibody incubation, proteins were detected using the ECL detection system (GE Healthcare, Buckinghamshire, UK) and exposed to Amersham Hyperfilm MP (GE Healthcare, Buckinghamshire, UK).

### Statistical analyses

To characterize the interaction between ionizing radiation and gossypol the combination index (CI) was calculated and isobolographic analysis was performed. The combination index was calculated according to the classic isobologram equation described by Chou and Talalay [32]:



In this equation, (D_x_)_1 _and (D_x_)_2 _represent the doses D_x _of compounds 1 and 2 alone required to produce an effect, and (D)_1 _and (D)_2 _represent isoeffective doses D when compounds 1 and 2 are given simultaneously. The combination index can either indicate additivity (CI = 1), synergism (CI < 1) or antagonism (CI > 1). For isobolographic analysis, full dose response curves of both gossypol and radiation were generated using Graph Pad Prism 4.0 software. From each combination effect classic isobolograms were constructed [33]. A combination point below the area of additivity indicated a synergistic interaction between both stimuli.

## Results

### Radiation and gossypol induce apoptosis

**Figure 2:**
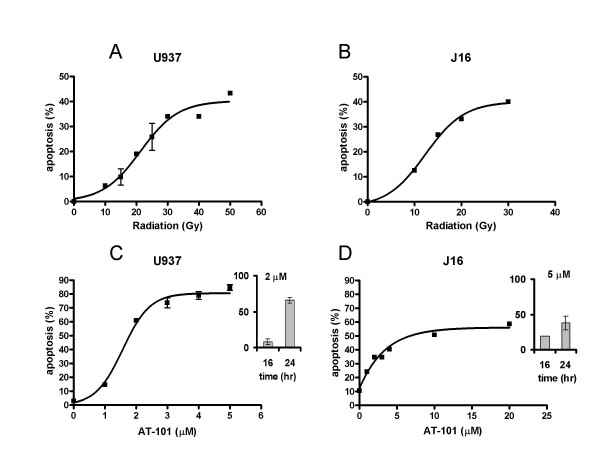
**Dose-dependent induction of apoptosis by radiation (A, B) and AT-101 (C, D) in human leukemic U937 (A, C) and Jurkat T cells (B, D)**. Apoptosis was quantified by FACScan analysis at t = 24 h after treatment. Data are presented as mean values (± SD) from 3 independent experiments. Inserts in C and D show the time-dependency of AT-101.

In both U937 and Jurkat T cells, radiation induced a time- and dose-dependent increase in apoptosis, measured by bisbenzimide staining and FACScan analysis, as reported previously [27,34,35]. The earliest morphological nuclear changes characteristic for apoptosis were detected after 6 h (not shown). Fig. 2A, B shows the dose-dependency of radiation-induced apoptosis in the two cell lines; ED_50 _values at t = 24 h are presented in Table 1.

**Table 1:** ED_50 _values for radiation and gossypol in human leukemic cells

	**U937**	**Jurkat T**
Radiation (Gy)	21.6	12.6
AT-101 (μM)	2.4	1.9
(±)-Gossypol (μM)	5.8	2.4

Values are derived from full dose-response curves for each stimulus at t = 24 h; data are mean values from 2 independent experiments.

Like radiation, AT-101 induced typical morphological features of apoptosis in a time- and dose-dependent fashion (Fig. 2C, D). As expected, AT-101 was more potent than the racemic mixture, which is reflected in the difference of their respective ED_50 _values (Table 1). AT-101-induced apoptosis was observed from 8 h onwards. Both radiation- and AT-101-induced apoptosis was fully inhibited by the pan-caspase inhibitor Z-VAD (data not shown).

### Interaction between radiation and AT-101 is synergistic and sequence-dependent

**Figure 3:**
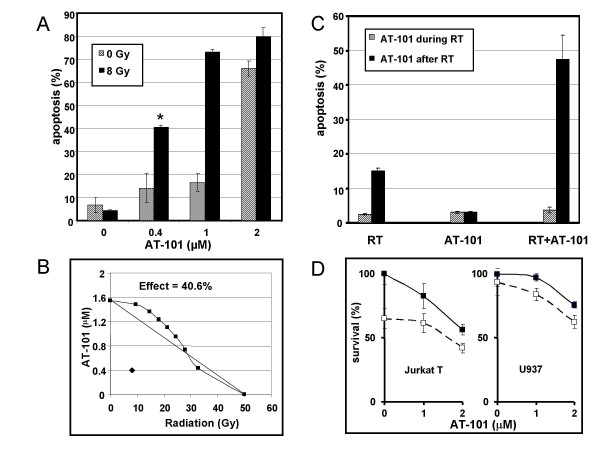
**Synergistic and sequence-dependent interaction between radiation and AT-101 in U937 cells**. A: The combination of radiation and AT-101 induces more apoptosis than the sum of the effects caused by the single agent treatment. Hatched bars represent the apoptotic effect by AT-101 alone (0-2 μM); black bars represent the combined effect with radiation (8 Gy). B: Isobolographic analysis of the combined effect of 40.6% apoptosis (* in A) induced by 0.4 μM AT-101 and 8 Gy radiation. The combination point is projected below the area of additivity, indicating synergy. The combination index for this point: CI = 0.42. C: Sequence-dependency of radiation and AT-101. Radiation (6 Gy) and AT-101 (1 μM) were either applied concurrently (hatched bars) or sequentially (AT-101 24 h after radiation; black bars). Apoptosis was analyzed at t = 24 h after AT-101. D: MTT cell viability assays in Jurkat T and U937 cells. AT-101 was added at the indicated concentrations (solid lines); radiation was dosed at 8 Gy (dashed line). Viability was determined at t = 48 h after radiation (i.e. 24 h after AT-101). Data presented in A, C and D are mean values (± SD) from 2 independent experiments.

To test the combined effect of both modalities, U937 and Jurkat T cells were irradiated with increasing doses of gamma rays (0-32 Gy) and 24 h later treated with different concentrations of AT-101 (0-10 μM). At various time points up to 24 h after treatment with AT-101, apoptosis was determined by propidium iodide staining and FACScan analysis. The combination of radiation and AT-101 induced more apoptosis than radiation alone and exceeded the sum of the effects caused by the single agent treatments (Fig. 3A). To characterize the type of interaction between both treatment modalities, the Combination Indices were calculated and isobolographic analyses were performed. For these calculations data from full dose-response curves were used. These tests revealed a clear synergistic interaction between radiation and AT-101, as illustrated by a Combination Index of 0.42 and a combined effect that is projected below the area of additivity in the isobologram (Fig. 3B).

To determine whether the observed combined effect was sequence-dependent as shown by others [22], sequential treatment (radiation followed by AT-101) was compared with concurrent delivery. As shown in Fig. 3C only when radiation was applied prior to AT-101 treatment, supra-additive levels of apoptosis were found. The interval between both modalities should at least be 16 h (not shown). In contrast, concurrent treatment did not result in significant interaction which is in agreement with previous observations [22].

In addition, the effect of AT-101 and radiation on cell viability was measured using the MTT assay under conditions where we showed apoptosis induction to be synergistic. Cells were first irradiated and 24 h later treated with AT-101. Cell viability was measured another 24 h later. As shown in Fig. 3D, AT-101 induced in a dose-dependent loss of viability, but did not further reduce cell survival after radiation.

### Gossypol and radiation activate the SAPK/JNK pathway

**Figure 4:**
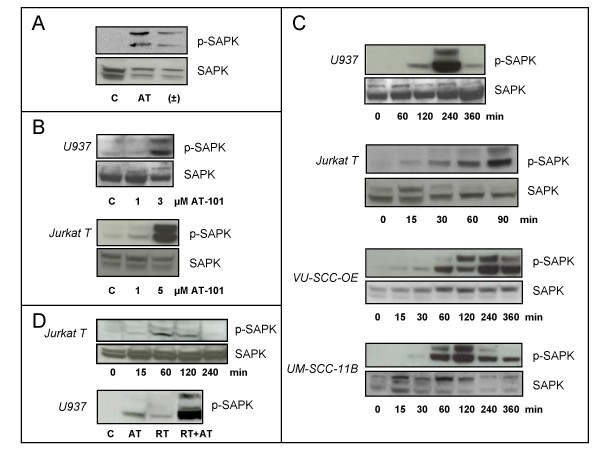
**Gossypol and radiation activate the SAPK/JNK pathway**. A: AT-101 is a stronger activator of SAPK/JNK than racemic (±)-gossypol. U937 cells were treated with equimolar concentrations of AT-101 (5 μM) and SAPK/JNK activation was analyzed at t = 2 h. (Abbreviations: C = control; AT = AT-101; ± =(±)-gossypol). B: Dose-dependent SAPK/JNK activation in U937 (upper panel) and Jurkat T cells (lower panel). Cells were treated with indicated concentrations of AT-101 and SAPK/JNK activation was analyzed at t = 2 h. C: Kinetics of 5 μM AT-101-induced SAPK/JNK in human leukemic (U937 and Jurkat T) and carcinoma cells (VU-SCC-OE and UM-SCC-11B). D: Radiation (8 Gy) induces a time-dependent SAPK/JNK activation in Jurkat T cells (upper panel). In U937 cells, the combination of AT-101 (AT; 5 μM) and radiation (RT; 10 Gy) induces a stronger activation of SAPK/JNK at t = 2 h than single modality treatment (lower panel).

Because SAPK/JNK-mediated signaling plays an important role in radiation-, chemotherapy- and environmental stress-induced apoptosis [27,34], we tested whether gossypol also activates this signaling pathway. As shown in Fig. 4A and consistent with the apoptosis-inducing capacity, AT-101 is a more potent activator of SAPK/JNK than racemic gossypol at equimolar concentrations. SAPK/JNK is activated by AT-101 in a dose- and time-dependent manner (Fig. 4B and 4C) in a variety of human tumor cell lines, including leukemic (U937, Jurkat T) and carcinoma cells (VU-SCC-OE, UM-SCC-11B). As illustrated in Fig. 4C, the kinetics of AT-101-induced SAPK/JNK activation varied among these different cell lines. The earliest response was observed around 15 min. after treatment. Fig. 4D shows the time-dependent activation of SAPK/JNK by radiation in Jurkat T cells and illustrates the strongly enhanced SAPK/JNK response after combined treatment with radiation and AT-101 in U937 cells.

**Figure 5:**
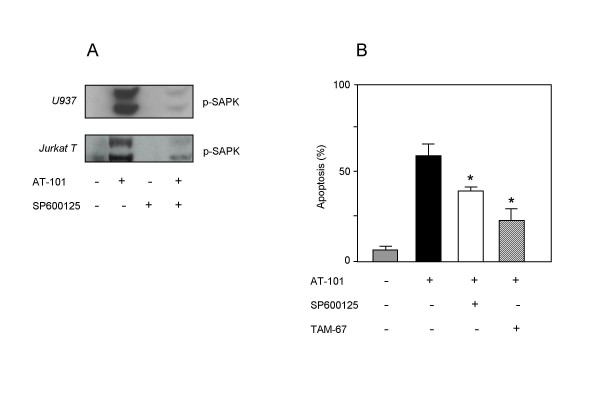
**AT-101 employs the SAPK/JNK pathway to induce apoptosis**. A: AT-101 (5 μM) induced SAPK/JNK in U937 and Jurkat T cells can be inhibited by the SP600125 kinase inhibitor; t = 90 min. B: Blockade of SAPK/JNK signaling by kinase inhibitor (SP600125) or dominant-negative c-Jun (TAM-67) inhibits AT-101 (5 μM)-induced apoptosis at t = 20 h in U937 cells. Data are presented as mean values (± SD) from 2 independent experiments. *p < 0.005, Student's *t *test.

To assess the role of the SAPK/JNK pathway in AT-101-induced apoptosis, we used the kinase inhibitor SP600125 [30] and the c-Jun dominant-negative deletion mutant TAM-67 [31] in U937 cells. As shown in Fig. 5A, SP600125 inhibited AT-101-induced SAPK/JNK activation in both cell types studied, while the compound itself had no effect. Treatment with SP600125 also significantly reduced AT-101-induced apoptosis (Fig. 5B). Moreover, in U937 cells stably expressing the dominant negative mutant of c-Jun, TAM-67, AT-101-induced apoptosis was significantly reduced as compared to vector-only controls. Taken together, these findings indicate a requirement for SAPK/JNK signaling in AT-101-induced apoptosis.

## Discussion

Overexpression of anti-apoptotic members of the Bcl-2 family is frequently observed in many different tumor types and has been associated with resistance to radio- and chemotherapy and poor prognosis. The identification of gossypol as an orally available, potent small molecule inhibitor of several anti-apoptotic members of the Bcl-2 family provides a rationally designed strategy to overcome this resistance and improve clinical outcome. In the present studies, we investigated the effect of AT-101 on radiation-induced apoptosis in human U937 and Jurkat T leukemic cells. We demonstrated that AT-101 strongly enhanced radiation-induced apoptosis to levels that exceeded additivity, as shown by isobolographic analysis. Furthermore, activation of the SAPK/JNK pathway, which is known to mediate radiation-induced apoptosis, was found to play an important role in the cytotoxic effects of AT-101.

Proteins of the Bcl-2 family mediate mitochondrial permeability and are therefore the key regulators of the intrinsic apoptotic pathways [36]. Bcl-2 proteins contain regions of amino acid sequence similarity, known as Bcl-2 homology (BH) domains. The family consists of the anti-apoptotic Bcl-2 group (such as Bcl-2, Bcl-X_L_, Mcl-1), the pro-apoptotic Bax group (Bax, Bak and Bok) and the pro-apoptotic BH3 domain-only group (including Bad, Bid, Noxa, Puma). Bcl-2 family members can homo- and heterodimerize. Dimerization and multimerization is essential for their function. Under normal conditions, BH3 domain-only proteins are either expressed at low levels or remain inactive in the cytoplasm. In response to a unique type of stress stimulus a BH3 domain-only protein is activated and translocates to the mitochondria to exert its pro-apoptotic effect. There are two models that describe how BH3 domain-only proteins work [36]. According to one model (the direct model), they transiently interact with Bax and/or Bak to induce their homomultimerization forming a pore through which cytochrome c and other apoptogenic mediators are released. Inhibitory Bcl-2 family members can bind and sequester BH3 domain-only molecules, thereby preventing their pro-apoptotic interaction with Bax or Bak. According to another model, the indirect model, Bax and Bak are complexed by inhibitory Bcl-2 family members. BH3 domain-only members release Bax and Bak from such inhibition by displacing them in the complex. In this way, Bax or Bak are also free to form the homomultimer and cause mitochondrial permeabilization.

Thus, the anti-apoptotic function of Bcl-X_L_/Bcl-2 is largely attributed to their ability to interact with pro-apoptotic members of the Bcl-2 family through the hydrophobic BH3 binding α helix, thereby preventing Bax/Bak-mediated release of cytochrome c. According to this mechanism, small molecules that interact with the BH3 binding α helix of Bcl-X_L_/Bcl-2 will function as Bcl-X_L_/Bcl-2 antagonists and promote apoptosis. In a search for such candidates, the combination of computer modeling and *in vitro *fluorescence polarization displacement studies demonstrated a direct inhibition of the binding between a 16-residue Bak BH3 peptide and Bcl-X_L _and Bcl-2 by gossypol with IC_50 _values of 0.4 μM and 10 μM, respectively [21]. Moreover, *in silico *docking studies using the 3-dimensional structure of Bcl-X_L _predicted gossypol to bind in the deep hydrophobic groove on the surface of Bcl-X_L _that is known to be the same site targeted by endogenous antagonists of this protein [15].

Gossypol has been shown to induce apoptosis in a variety of tumor cell lines overexpressing Bcl-X_L _and/or Bcl-2 [15,16,18]. In addition, an antitumor effect was shown in several cancer cell types [37-42]. Not many studies, however, have considered the cytotoxic effect of gossypol in combination with radio- and/or chemotherapy. In the human prostate cancer cell line PC-3, AT-101 potently enhanced radiation-induced apoptosis and growth inhibition and reduced clonogenic survival [22]. (±)-Gossypol induced enhanced radiosensitivity, albeit with substantial variation in a panel of carcinoma cell lines, which primarily resulted from reduced double-strand break repair capacity [43]. In lymphoma cells the addition of CHOP chemotherapy significantly enhanced AT-101-induced cytotoxicity [21].

In the present studies we show a dose- and time-dependent induction of apoptosis by AT-101 in two human leukemic cell lines. Consistent with the observation of others [44,45], the (-) enantiomer was more potent in inducing apoptosis than racemic gossypol as reflected by the ED_50 _values. In addition, AT-101 strongly enhanced radiation-induced apoptosis in a sequence-dependent fashion. The type of interaction between both stimuli was synergistic as demonstrated by isobolographic analysis and a combination index smaller than 1.0. The nature of this enhancing effect is unknown, but is clearly the result of partially overlapping and, more importantly, partially distinct mechanisms. Radiation is known to induce the apoptotic cascade via the mitochondria-dependent intrinsic pathway where cytochrome c release is the critical event leading to caspase activation. The major mode of action of gossypol is through its interaction with the BH3-binding groove in Bcl-X_L _and to a lesser extent in Bcl-2, thereby preventing their interaction with pro-apoptotic proteins and allowing mitochondrial permeabilization. In addition, AT-101 has been found to bind to and inhibit the anti-apoptotic function of Mcl-1 [46]. Gossypol may also directly interact with pro-apoptotic Bcl-2 family members (Bax, Bak) and promote their multimerization which is essential for the release of cytochrome c [19].

Because gossypol has been reported to also increase radiosensitivity [22,43], we generated clonogenic survival (data not shown) and cell viability curves, but could not detect significant radiosensitization. This indicates that in the cell systems used apoptosis is the prevailing mode of cell death after the combination of radiation and AT-101. Moreover, this short term cell kill could be fully inhibited by the pan-caspase inhibitor Z-VAD.

Activation of SAPK/JNK has been shown to be essential for apoptosis induction by many types of cellular stress, including radiation and chemotherapeutic drugs [27,47,48]. The SAPK/JNK pathway involves sequential phosphorylation and activation of the proteins MAPK/ERK kinase kinase 1, SAPK/ERK kinase 1, SAPK/JNK and c-Jun. There are several observations by others that prompted us to investigate the effect of gossypol on this pro-apoptotic signaling system. First, because overexpression of one of the prime targets of gossypol, Bcl-X_L_, was reported to inhibit SAPK/JNK [29], we reasoned that blocking this (and other) anti-apoptotic protein, the pro-death signaling would be restored. Second, it has been shown that SAPK/JNK translocates to the mitochondria upon irradiation and other stress factors where it phosphorylates and inactivates anti-apoptotic Bcl-2 family members, including Bcl-2, Bcl-X_L _and Mcl-1 [49-51]. Finally, other investigators have recently shown that Bcl-2 antagonists like gossypol, can increase bortezomib-mediated cellular stress and SAPK/JNK activation in lymphoma cells [52]. We have previously shown that stimulation of the SAPK/JNK pathway is essential for radiation-induced apoptosis in both J16 and U937 cells [34,46]. In our present studies, we found that in both leukemic cells and squamous cell carcinoma gossypol rapidly activated the SAPK/JNK pathway, notably with AT-101 being more effective than the racemic (±)-gossypol. Importantly, activation of SAPK/JNK preceded the appearance of the typical morphological features of apoptosis, indicating a temporal relation between both events. The pivotal role of SAPK/JNK in AT-101-induced apoptosis was demonstrated by our experiments using the SAPK/JNK inhibitor SP600125 and the dominant-negative mutant of c-Jun. This mutant, denominated TAM-67, lacks the N-terminal transactivation domain of c-Jun, including Ser-63 and Ser-73, the sites of phosphorylation and activation of the SAPK/JNK pathway [31]. SP600125 significantly inhibited AT-101-induced SAPK/JNK phosphorylation and apoptosis induction. Moreover, in cells overexpressing the TAM-67 mutant, AT-101-induced apoptosis was significantly reduced. Collectively, these data suggest that not only radiation-, but also AT-101-induced apoptosis requires a functional SAPK/JNK signaling system.

## Conclusion

In summary, we have demonstrated that AT-101 strongly enhances radiation-induced apoptosis to supra-additive levels. We present evidence that activation of the SAPK/JNK pathway significantly contributes to the apoptotic effect of AT-101. This combined approach represents an attractive strategy to overcome treatment resistance due to overexpression of anti-apoptotic Bcl-2 family members. We are currently performing preclinical proof-of-principle studies with this novel combined modality treatment in a mouse xenograft tumor model.

## Competing interests

The authors declare that they have no competing interests.

## Authors' contributions

SFZ carried out the apoptosis and MTT assays, Western blotting and statistical analyses and participated in the design of the study. RS carried out part of the Western blotting. GK, DY, MEL, WJB, HB and VL participated in the design of the study and analyzed data. RR carried out part of the apoptosis assays and provided supplementary results. MV conceived and designed the experiments, analyzed data and wrote the paper. All authors read and approved the final manuscript.
